# Influenza epidemiology and burden of disease in Mongolia, 2013–2014 to 2017–2018

**DOI:** 10.5365/wpsar.2020.11.4.003

**Published:** 2021-06-07

**Authors:** Oyungerel Darmaa, Alexanderyn Burmaa, Baataryn Gantsooj, Badarchiin Darmaa, Pagbajabyn Nymadawa, Sheena G Sullivan, James E Fielding

**Affiliations:** a National Influenza Centre, National Centre of Communicable Diseases, Ulaanbaatar, Mongolia.; b Mongolian Academy of Medical Sciences, Ulaanbaatar, Mongolia.; c World Health Organization Collaborating Centre for Reference and Research on Influenza, Royal Melbourne Hospital, at the Peter Doherty Institute for Infection and Immunity, Melbourne, Australia.; d Melbourne School of Population and Global Health, University of Melbourne, Melbourne, Australia.; e Victorian Infectious Diseases Reference Laboratory, Royal Melbourne Hospital, at the Peter Doherty Institute for Infection and Immunity, Melbourne, Australia.

## Abstract

**Background:**

Mongolia is a vast, sparsely populated country in central Asia. Its harsh climate and nomadic lifestyle make the population vulnerable to acute respiratory infections, particularly influenza. Evidence on the morbidity, mortality and socioeconomic impact of influenza in Mongolia is scarce; however, routine surveillance for influenza-like illness (ILI), severe acute respiratory infection (SARI) and laboratory-detected influenza is conducted. This paper describes the epidemiology of influenza and the estimated burden of influenza-associated illness in Mongolia in the five influenza seasons between 2013–2014 and 2017–2018.

**Methods:**

Demographic and laboratory data from 152 sentinel surveillance sites on all patients who met the case definitions of ILI and SARI between October 2013 and May 2018 were extracted and analysed as described in *A Manual for Estimating Disease Burden Associated with Seasonal Influenza*.

**Results:**

The estimated annual influenza-associated ILI and SARI rates, presented as ranges, were 1279–2798 and 81–666 cases per 100 000 population, respectively. Children aged < 5 years accounted for 67% of all ILI cases and 79% of all SARI cases. The annual specimen positivity for influenza was highest (11–30% for ILI and 8–31% for SARI) for children aged 5– < 15 years and children < 2 years old, respectively. The annual mortality rate due to pneumonia and SARI was highest among children aged < 2 years (15.8–54.0 per 100 000 population). Although the incidence of influenza-associated ILI and SARI was lowest for people aged ^3^65 years, the mortality rate due to pneumonia and SARI (1.2–5.1 per 100 000) was higher than that for those aged 15–64 years.

**Conclusion:**

The estimated influenza-associated ILI and SARI incidence rates are high in Mongolia, and children, especially those aged < 5 years, have the highest influenza-associated burden in Mongolia. These findings provide evidence for decision-makers in Mongolia to consider targeted influenza vaccination, particularly for children.

Influenza is a highly infectious acute respiratory disease that is estimated to result globally in 3–5 million cases of severe illness and 290 000–650 000 deaths annually. (*1*, *2*) Children aged < 5 years are more susceptible to infection, with an estimated annual attack rate of 20–30%, compared with adults at 5–10%, with the elderly having the highest risk of mortality. (*3*)

Syndromic and virological surveillance of influenza-like illness (ILI) and severe acute respiratory infection (SARI) are used to understand and estimate the burden of influenza. The data generated can be used to identify populations at high risk of infection and of complications, provide early warning of potential epidemics and guide preparedness, resource allocation, selection of preventive, treatment and control measures and selection of strains for seasonal flu vaccination. (*4*) In developing, low-income countries such as Mongolia, however, the burden of influenza is poorly quantified. (*5*, *6*) In 2015, the World Health Organization (WHO) published *A Manual for Estimating Disease Burden Associated with Seasonal Influenza* to guide comparable studies of disease burden with a uniform method. (*7*) The method is based on available data from national surveillance that countries may use annually and will result in comparable results across time and geography if the surveillance methods are applied consistently.

Mongolia is a landlocked country in east and central Asia. The population of about 3 million people is relatively young with 65% aged < 35 years. Children aged < 5 years and people aged > 65 years constitute 13% and 4% of the population, respectively. (*8*) The temperature ranges from approximately –30 °C to 40 °C, and the capital, Ulaanbaatar, in which nearly half the population resides, is considered the coldest capital city in the world. (*9*) One third of the population resides in rural areas, breeding livestock in nomadic and semi-nomadic pastoralism. The country’s harsh climate and nomadic lifestyle make the population vulnerable to acute respiratory infections, particularly influenza. (*10*)

Several studies of sentinel surveillance of ILI and SARI and a cohort study conducted in a district family general practice 130 km east of Ulaanbaatar in 2010–2011 showed that, between 2007–2008 and 2011–2012, children aged < 5 years had the highest incidence, accounted for almost all cases of ILI and SARI and had the highest attack rate of laboratory-confirmed influenza. (*11*-*13*) In this study, we sought to further elucidate the burden of influenza in Mongolia. Syndromic and laboratory surveillance data were used to compare morbidity and mortality, and to estimate the disease burden associated with seasonal influenza with the standardized protocol described in the WHO manual, in Mongolia between 2013–2014 and 2017–2018.

## Methods

### Health facilities in Mongolia

Administratively, Mongolia is divided into nine districts in the capital, Ulaanbaatar, and 21 provinces; the provinces are divided into subregions called soums. Outpatients are managed in the capital districts in 218 family health centres and in the provinces and soums in 296 soum health centres. Data on ILI are collected from all outpatient sites in the country and reported weekly to the nine district health departments in the capital and 21 provincial health departments and then forwarded to the National Centre for Communicable Diseases (NCCD). The data collected and reported to the flu information system (www.flu.mn) consisted of the total number of outpatient visits to family group practices, the total number of ILI cases, the number of clusters, the total number of ambulance calls and the total number of calls due to ILI.

The largest hospitals in the country are general hospitals, regional centres for diagnosis and treatment and specialized hospitals known as reference centres. There are general hospitals in each of the capital districts and in 16 provinces and regional centres for diagnosis and treatment in five provinces. Although all public health facilities participate in influenza surveillance, cases diagnosed and treated in private hospitals are not reported. The proportions of patients treated in public and private facilities were not available.

### Sentinel surveillance

The WHO case definitions of ILI and SARI were used. An ILI case was defined as an acute respiratory infection with measured fever of  > 38 °C and cough with onset within the previous 10 days. (*4*) A SARI case was defined as an acute respiratory infection with a history of fever or measured fever of  > 38 °C and cough with onset within the previous 10 days and requiring hospitalization. (*4*)

ILI surveillance with specimen collection has been conducted at 115 sentinel outpatient sites throughout the country (23 in the capital and 92 in the provinces) since 2009. The sites report data on ILI daily. The sampling and testing methods are described under “Specimen collection and testing” below. SARI surveillance has been conducted at 37 hospitals since 2009, of which 16 are provincial general hospitals (located in provincial capital cities), five are regional centres for diagnosis and treatment, nine are district general hospitals in the capital, three are soum hospitals (one in the coldest part of the country, one in the south close to a major border crossing with China and the other in the north close to a major border crossing with the Russian Federation) and four are reference centres (National Centre of Maternal and Child Health, National Cancer Centre, State Hospital Number 3 and the NCCD hospital). The hospitals report data on SARI inpatients once a week. The data collected and reported to the flu information system consisted of the total number of patients at the end of the previous week, the total number of recovered and shifted patients, deaths, total number of newly admitted patients, number of patients at the end of the current week, total number of SARI patients at the end of the previous week, total number of recovered and shifted patients, deaths, total number of newly admitted SARI patients and total number of SARI patients at the end of the current week.

### Specimen collection and testing

The sentinel surveillance sites are classified into one of two categories according to the frequency of specimen collection. Category I sites (*n* = 78: 61 outpatient sites and 17 hospitals) collect and send specimens for testing every week, and category II sites (*n* = 74: 54 outpatient sites and 20 hospitals) collect and send specimens for testing only during the influenza season or if an outbreak or cluster is detected at the site.

Physicians at ILI and SARI sentinel sites were asked to collect nasopharyngeal swabs each week from 5–10 patients who met the case definitions within 3 days of disease onset and before treatment. The collected specimens were immediately immersed into sterile tubes containing virus transport medium, stored in refrigerators at the sentinel sites and transported to the Reference Virology Laboratory of the National Influenza Centre at the NCCD or to one of four participating branch laboratories. (*12*) Samples were shipped by car from the central region, by plane from the western and eastern regions and by train from the northern and south-eastern regions. Samples were tested for influenza virus by real-time reverse transcriptase polymerase chain reaction. Virus-positive samples were passaged in MDCK cells for isolation. Genetic sequencing analysis was done for five strains of A(H1N1)pdm09 and eight strains of A(H3N2) by ABI Big Dye terminator v.3.1 Cycle Sequencing and ABI 3130 xl Analyser.

### Analysis of epidemiological data and burden of disease

Data from the ILI, SARI and laboratory surveillance systems in the 2013–2014 and 2017–2018 influenza seasons were analysed to elucidate the epidemiology of influenza in Mongolia. As the annual influenza season crosses the new calendar year, seasons were defined as from week 40 of one year to week 39 of the following year. ILI incidence rates were calculated from population data for the whole country and total consultations and for the populations of the capital city districts and provincial capital cities. (*8*) SARI incidence rates were calculated from total hospitalizations and the populations of the provincial capitals and the districts of the national capital, representing the catchment populations of the SARI sentinel sites. An influenza season was defined as the period between the date on which the ILI rate crossed the median weekly ILI threshold rate for 2013–2014 to 2017–2018. Laboratory data were analysed by influenza type or subtype and the percentage of specimens tested that were positive for influenza.

These data were used to estimate overall and age-specific (age groups: < 2, 2– < 5, 5– < 15, 15– < 50, 50– < 65, and (*3*)65 years) influenza-associated medically attended ILI and SARI incidence rates for each season between 2013–2014 and 2017–2018, as described in WHO’s *A Manual for Estimating Disease Burden Associated with Seasonal Influenza*. (*10*)

Population mortality and case fatality rates were estimated from the ILI and SARI surveillance data collected throughout the season. Influenza-associated mortality and case fatality rates were not estimated, as samples were not taken from all people who died from SARI. Microsoft Excel® 2013 software was used for the data analyses.

## Results

**Figure 1:**
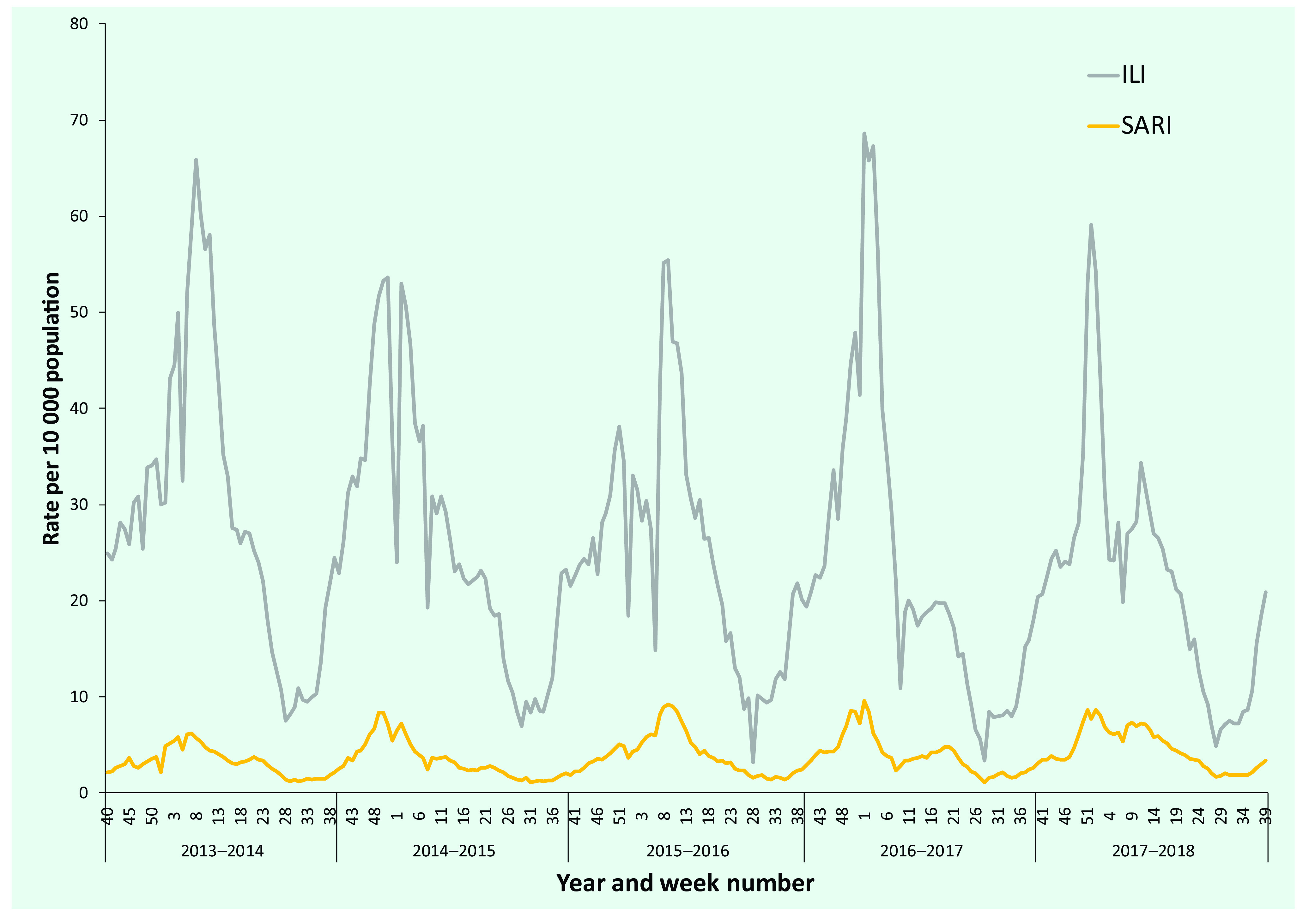
ILI and SARI rates per 10 000 population by week, Mongolia, 2013–2014 to 2017–2018

Between 2013–2014 and 2017–2018, 2 002 825 patients with ILI and 205 991 with SARI were reported per year (ranges, 371 491–440 389 for ILI and 33 136–50 759 for SARI). The seasonal peak rates were 50–70 ILI cases and 6–10 SARI cases per 10 000 population (**Fig. 1**). The peak rates were highest for both ILI and SARI in the 2016–2017 season, while the lowest peaks were in 2014–2015 and 2013–2014, respectively.

[**insert** Figure 1]

During the five seasons, the number of SARI patients increased, as both a proportion of hospital admissions and incidence rate, while the number of ILI patients decreased as a proportion of consultations and incidence rate (**Table 1**). The 5-year averages were 532.4 ILI cases and 42 SARI cases per 100 000 population.

**Table 1:** ILI consultations and SARI hospitalizations, incidence rates and season characteristics by year, Mongolia, 2013–2014 to 2017–2018

-	Influenza season					5-year average
	2013–2014	2014–2015	2015–2016	2016–2017	2017–2018	
ILI cases per total outpatient consultations (%)	5.7	5.2	4.7	4.5	4.4	4.9
No. of ILI cases per 100 000 population	15 029	13 732	13 102	12 145	11 690	13 106
SARI patients among total hospitalizations (%)	8.4	9.2	10.3	11.1	11.8	10.2
No. of SARI cases per 100 000 population	1660	1780	2079	2029	2364	1990
Season onset (week)	40	41	42	44	43	
Season peak (week)	8	51	9	1	52	
Season end (week)	22	13	19	7	16	

**Figure 2:**
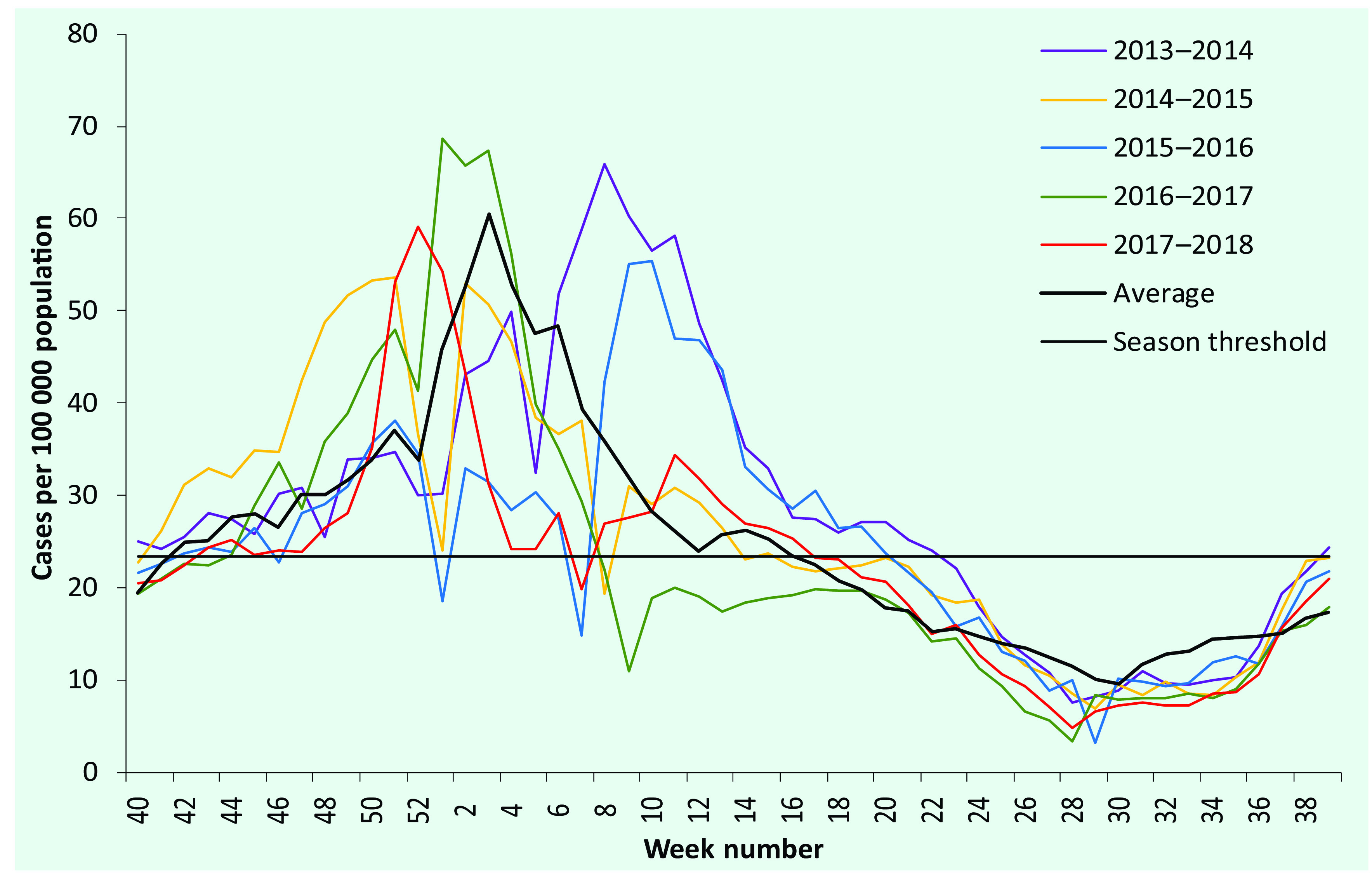
ILI rates by week and year, Mongolia, 2013–2014 to 2017–2018

Seasons started between weeks 40 and 44, but the timing of the peaks (weeks 51 to 9) and the ends (weeks 7 to 22) varied more widely (**Table 1**, **Fig. 1** and **2**). The longest season was that of 2013–2014 (35 weeks), and the shortest was that of 2016–2017 (16 weeks).

**Figure 3:**
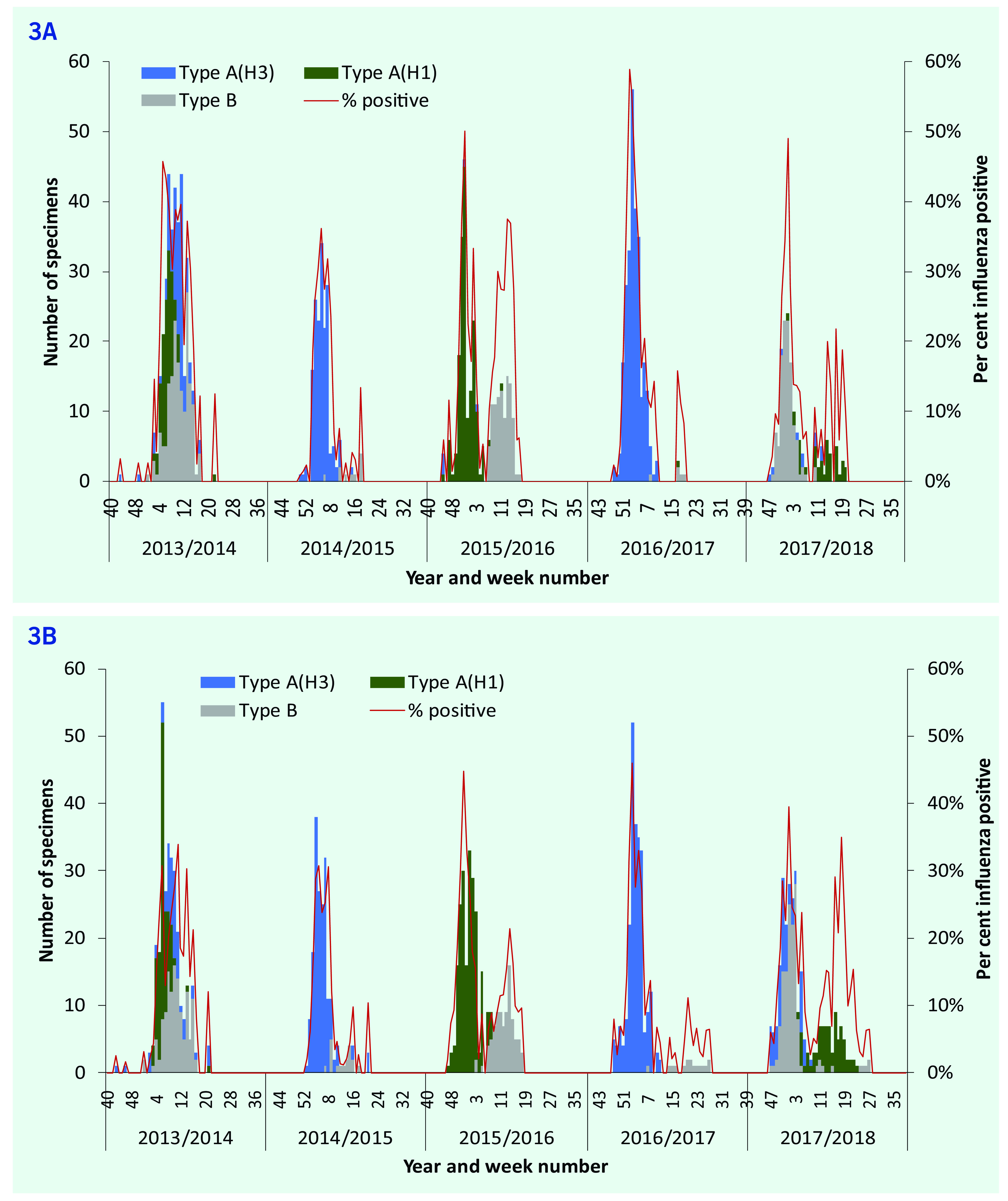
Numbers of ILI cases (3A) and SARI cases (3B) positive for influenza by type or subtype and percentages influenza positive, Mongolia, 2013–2014 to 2017–2018

Influenza virus was detected in the population in each of the five seasons of 2013–2014 to 2017–2018. Two seasons (2013–2014 and 2014–2015) started earlier, with most cases detected in weeks 2–17, peaking in weeks 3–11 (**Fig. 3**). Most cases in seasons 2015–2016, 2016–2017 and 2017–2018 were detected in weeks 47–17, with peaks in weeks 51–5. Trends in influenza positivity were similar for ILI and SARI patients. The 2014–2015 and 2016–2017 seasons were dominated by type A(H3N2), while type B co-circulated with type A(H1N1) in 2014–2015 and 2017–2018, with two distinct peaks in each season. All three types or subtypes co-circulated in 2013–2014. The percentage of tests positive for influenza virus was highest for both ILI and SARI patients in 2016–2017; however, over each full year, there was more variation in the percentage of ILI patients positive for influenza (range: 9–19%; lowest in 2014–2015 and highest in 2013–2014) than of SARI patients (range: 8–12%; lowest in 2014–2015 and highest in 2017–2018).

[**insert** Figure 2]

[**insert** Figure 3]

The highest proportions of the population positive for both ILI and SARI from 2013–2017 to 2017–2018 were reported in children aged < 5 years (**Tables 2** and **3**). In seasons 2015–2016, 2016–2017 and 2017–2018, for which fewer data were available by age group, the proportions were highest among those aged < 2 years. The proportions of age group-specific ILI generally decreased with age, whereas for SARI, the proportions decreased with age to 50 years and then increased for older age groups.

**Table 2:** Range of annual ILI cases, incidence and per cent influenza positive by age group, 2013–2014 to  2017–2018

-	Age group (years)								Total
	< 2*	2– < 5*	< 5	5– < 15	15– < 50	^3^50	50– < 65*	^3^65*	
Total outpatient consultations	1 228 239– 1 354 384	765 072– 804 931	1 955 342– 2 180 652	877 738– 1 086 993	2 632 829– 3 666 852	1 714 868– 2 135 206	1 117 744– 1 322 442	721 168– 812 764	7 734 051– 8 502 958
ILI cases	148 950– 174 258	100 979– 104 964	249 929– 285 412	71 670– 101 315	34 472– 41 364	9867– 18 261	6170–7792	3727–4133	371 491– 440 389
ILI cases per 100 000 population	96 575– 108 028	42 118– 46 535	63 436– 87 486	13 097– 20 981	2007–2411	2208–4430	1852–2108	3239–3368	11 690– 15 029
ILI cases sampled	488–638	495–651	667–1289	295–736	230–417	76–380	55–68	17–32	1615–2447
% specimens influenza positive	10–13	14–18	6–15	11–30	9–19	9–24	9–25	10–18	9–19
Estimated% total consultations for influenza-associated ILI	1.3–1.6	1.8–2.5	0.8–1.9	0.9–2.8	0.1–0.3	0.0–0.1	0.1–0.1	0.0–0.1	0.5–1.1
Estimated influenza-associated ILI per 100 000 population	10 498– 13 476	5786–8435	4958– 10 079	1642–6313	191–457	203–618	168–500	308–582	1279–2798

* For seasons 2015–2016, 2016–2017 and 2017–2018 only

**Table 3:** Range of annual SARI cases, incidence and per cent influenza positive by age group, 2013–2014 to 2017–2018

-	Age group (years)								Total
	< 2*	2– < 5*	< 5	5– < 15	15– < 50	^3^50	50– < 65*	^3^65*	
Total admissions	54 095– 66 759	20 389– 28 077	72 470– 87 148	20 209– 49 594	154 586– 204 850	105 027– 122 039	62 124– 69 403	41 738– 52 636	384 711– 432 053
SARI cases	25 018– 27 392	9724–12 640	26 476– 40 032	3003–6103	1391–2,654	1043– 1961	758–1034	621–927	33 136– 50 750
SARI cases per 100 000 population	23 315– 26 197	6359– 7 777	12 226– 14 988	894– 1 569	120–228	344–588	336–413	781–1114	1567–2,356
SARI cases sampled	756–804	434–563	1190– 1 725	124–209	66–337	17–119	13–51	4–25	1397–2390
% specimens influenza positive	8–31	11–30	4–30	12–22	4–17	4–21	8–16	5–50	5–28
Estimated% total hospitalizations for influenza- associated SARI	3.3–15.5	4.9–13.4	1.5–14.8	1.2–4.0	0.0–0.2	0.0–0.3	0.1–0.2	0.5–0.9	0.5–3.3
Estimated influenza- associated SARI per 100 000 population	1798– 8 039	697–2311	530–4547	103–316	8–23	14–104	32–57	65–557	81–666

* For seasons 2015–2016, 2016–2017 and 2017–2018 only

The estimated annual influenza-associated ILI and SARI rates, presented as ranges, were 1279–2798 and 81–666 cases per 100 000 population, respectively. (**Tables 2** and **3**) The rates were highest for children aged < 5 years (especially those aged < 2 years) and lowest for people aged 15– < 50 years. There was wider variation between the minimum and maximum annual rates of SARI (42%) than for ILI (29%). The annual rates of ILI decreased each year during the study period, while the rates of SARI increased.

The annual mortality rate due to SARI ranged from 1.2 to 3.9 deaths per 100 000 population between 2013–2014 and 2017–2018. The rate was highest among children aged < 5 years, in particular those aged < 2 years (15.8–54.0 deaths per 100 000 population in 2015–2016 to 2017–2018). Annual mortality rates were < 1.0 deaths among people aged 5–50 years, increasing to 1.2–5.1 deaths per 100 000 population for those aged (*3*)65 years (**Table 4**).

**Table 4:** Range of annual deaths and rates of mortality due to pneumonia and SARI, 2013–2014 to 2017–2018

-	Age group (years)								Total
	< 2*	2– < 5*	< 5	5– < 15	15– < 50	^3^50	50– < 65*	^3^65*	
Deaths due to SARI	17–59	4–6	21–65	1–2	0–5	1–8	1–4	1–4	25–80
Mortality rate per 100 000 population	15.8–54.0	2.5–3.9	7.9–24.8	0.3–0.6	0.0–0.4	0.3–2.6	0.4–1.8	1.2–5.1	1.2–3.9

* For seasons 2015–2016, 2016–2017 and 2017–2018 only

## Discussion

In this first study of the influenza burden in Mongolia, estimated with WHO’s *A Manual for Estimating Disease Burden Associated with Seasonal Influenza*, (*7*) the burden of influenza-associated ILI and SARI was highest among children aged < 5 years, especially among those aged < 2 years, consistent with a study conducted with the same methods on the epidemiology and impact of influenza in Mongolia between 2007 and 2012. (*13*) The estimated annual influenza-associated ILI and SARI rates, presented as ranges, were 1279–2798 and 81–666 cases per 100 000 population, respectively; the rates in children aged < 5 years were 4958–10 079 and 530–4547 per 100 000 population, respectively. These rates are higher than those in other published studies of influenza in low- and middle-income countries (LMICs), as classified by The World Bank. (*14*) For example, the influenza-associated SARI rates per 100 000 population for all ages and for children aged < 5 years, respectively, were: 115–142 and 2021–2349 in China; (*15*) 13–19 and 82–114 in Indonesia; (*16*) 21–82 and 147–469 (in children aged < 2 years) in Kenya; (*17*) and 43.9 and 187.7 in Zambia. (*18*) The studies should be compared cautiously, as the same (WHO) SARI case definition was used only in Indonesia and Kenya, and the rates were from a relatively small number of hospitals and extrapolated to provincial or national levels.

The outcomes of influenza may be more severe in LMICs than in high-income countries, particularly in pregnant women, people living with HIV/AIDS and children aged < 5 years, (*19*) contributing to a disproportionate proportion of the global burden of influenza. (*6*) There are several possible explanations for the very high rates observed in Mongolia. The extreme winter results in increased occupation of indoor spaces and may reduce immunity in some population groups. Increased population mixing also occurs in winter during public holidays, particularly the Lunar New Year and the beginning of the school year. Smoke and pollution caused by burning coal may exacerbate respiratory conditions and increase vulnerability to influenza infection.

The wide range of annual estimates of influenza-associated SARI in particular is partly driven by the marked, consistent increase in annual SARI rates over the 5-year study period. The reason for this increase has not been established, but hospitals may have changed the diagnostic and admission criteria for SARI to maximize government assistance payments for admitted SARI cases.

Only limited quantities of influenza vaccine are available in Mongolia, provided by the Government and the Partnership for Influenza Vaccine Introduction programme (https://pivipartners.org/). The Government subsidized influenza vaccination for health-care workers and staff in emergency agencies following the influenza A(H1N1)pdm09 pandemic in 2009, but vaccination remains voluntary and requires payment by other groups, so that very few people are vaccinated each year. The high influenza-associated ILI and SARI burden and mortality from pneumonia and SARI in children indicate that a vaccination programme for children could have an enormous impact on the burden of influenza in Mongolia. It would require considerable funding and resources in view of the high proportion of youth in the population. This population structure is common in developing countries, where 99% of deaths attributable to influenza-associated acute lower respiratory infection deaths in children aged < 5 years occur. (*20*)

In the five influenza seasons between 2013–2014 and 2017–2018, ILI and SARI activity in Mongolia usually started in October and peaked during the coldest period of the year between late December and February. As measured by the percentage of samples from ILI and SARI patients who tested positive for influenza, the highest seasonal load was in 2016–2017 and the lowest in 2014–2015. In the 2015–2016 and 2017–2018 seasons, distinct secondary peaks were seen, associated with other influenza types and subtypes that dominated later in the seasons. The subtype distribution was consistent in the ILI and SARI surveillance systems each year, influenza A(H3) being the predominant circulating subtype in 2014–2015.

The timing and distributions of type and subtype in each of the five influenza seasons varied during the surveillance period and were not always consistent with observations from other regions of the northern hemisphere. Between 2013–2014 and 2016–2017, the subtype distribution in Mongolia was similar to those of North America and of north and east Asia (particularly China, Japan and the Republic of Korea) in each of the four seasons and to that of Europe in three seasons; (*21*-*24*) however, the timing of the seasons was similar to those of these regions in only two of the four seasons. In 2017–2018, the timing and subtype distribution best matched that observed in western Europe. (*25*) The difference between the seasonal pattern in Mongolia and those in other countries in north and east Asia and elsewhere in the northern hemisphere highlights the importance of national surveillance in understanding influenza epidemiology and virology in Mongolia.

Our study had several limitations. A high staff workload, limited availability of swab kits, the absence of systematic sampling and the logistical challenges of transporting samples over long distances may have resulted in non-random sampling of ILI and SARI patients. The influenza-associated SARI mortality rate could not be estimated, as not all deaths were confirmed in the laboratory according to surveillance procedures. The positivity rate for influenza virus might have been underestimated due to delayed health care-seeking because of insufficient health literacy and improper use of antibiotics, lengthening the time to presentation to a doctor. Furthermore, reluctance among the elderly to seek health care may have resulted in underestimates of influenza-associated ILI, SARI and mortality rates for this age group. In contrast, SARI rates in the provinces might have been overestimated, as serious cases in soum hospitals are sometimes referred to provincial general hospitals. Lack of more detailed epidemiological and clinical data on cases prevented in-depth analysis of risk factors, such as underlying conditions and geographical distribution.

The estimated incidence of influenza-associated ILI and SARI in Mongolia over five seasons between 2013–2014 and 2017–2018 was higher than that in comparable countries; however, our finding that children under 5 years were the most affected is consistent with regional and global trends. The findings can inform influenza control policies. Targeted vaccination of children would dramatically decrease the burden of influenza in Mongolia. Further improvements to the surveillance system would allow more detailed analysis of risk factors and underlying conditions associated with the severity and economic burden of influenza.
